# The predictive value of cumulative atherogenic index of plasma (AIP) for cardiovascular outcomes: a prospective community-based cohort study

**DOI:** 10.1186/s12933-024-02350-8

**Published:** 2024-07-18

**Authors:** Zhihao Liu, Long Zhang, Leyi Wang, Kaiyin Li, Fangfang Fan, Jia Jia, Jianping Li, Yan Zhang

**Affiliations:** 1 Department of Cardiology, Peking University First Hospital, No. 8 Xishiku St, Xicheng District, Beijing, 100034 China; 2 Institute of Cardiovascular Disease, Peking University First Hospital, Beijing, China; 3 State Key Laboratory of Vascular Homeostasis and Remodeling, Peking University and NHC Key Laboratory of Cardiovascular Molecular Biology and Regulatory Peptides, Beijing, China

**Keywords:** Atherogenic index of plasma, Major adverse cardiac events, Stroke, Myocardial infarction, Death

## Abstract

**Background:**

Atherogenic index of plasma (AIP) has been reported as a critical predictor on the risks and clinical outcomes of cardiovascular diseases (CVDs), and we aimed to explore the potential predictive value of cumulative AIP on major adverse cardiac events (MACE), stroke, myocardial infarction (MI) and cardiovascular mortality.

**Methods:**

A large-scale community-based prospective cohort was established from December 2011 to April 2012 and followed up in May to July 2014. The endpoint outcomes were obtained before December 31, 2021. AIP was calculated as the logarithmically transformed ratio of triglyceride (TG) to high-density lipoprotein cholesterol (HDL-c) and cumulative AIP was the average value of AIP in 2012 and 2014.

**Results:**

An overall of 3820 participants (36.1% male) with mean (SD) age of 59.1 (8.7) years, were enrolled. Within a median follow-up of 7.5 years, a total of 371 (9.7%) participants were documented with MACE, 293 (7.7%) participants developed stroke, 68 (1.8%) suffered from MI and 65 (1.7%) experienced cardiovascular mortality. Multivariable Cox regression analysis revealed significant associations between cumulative AIP and the risk of MACE, stroke and MI. Regarding MACE, individuals with one higher unit of cumulative AIP were associated with 75% increment on the incidence of going through MACE in fully adjusted model, while categorizing participants into four groups, individuals in the highest cumulative AIP quartile were significantly associated with increased incidence of MACE (HR = 1.76, 95%CI: 1.27–2.44, *p* < 0.001 in fully adjusted model), stroke (HR = 1.69, 95%CI: 1.17–2.45, *p* = 0.005) and MI (HR = 2.82, 95%CI: 1.18–6.72, *p* = 0.019). But not a significant association was observed between cumulative AIP and cardiovascular mortality. In subgroup analysis, the association of cumulative AIP and the incidence of stroke was more pronounced in the elderly (HR: 0.89 vs. 2.41 for the age groups < 65 years and ≥ 65 years, *p* for interaction = 0.018).

**Conclusions:**

A higher cumulative AIP was significantly associated with an increased risk of MACE, stroke and MI independent of traditional cardiovascular risk factors in a community-based population, and the association of cumulative AIP and stroke was particularly pronounced in the elderly population.

**Supplementary Information:**

The online version contains supplementary material available at 10.1186/s12933-024-02350-8.

## Introduction

Cardiovascular disease (CVD) is a prevalent and debilitating condition that causes substantial economic and social burdens worldwide [1, 2]. Novel indicators have been proposed in the attempt to fill the gap of residual risks left unassessed by traditional CVD risk factors [3–7].

Atherogenic index of plasma (AIP), defined as a logarithmic transformation of the ratio of the molar triglyceride (TG) concentration and high-density lipoprotein cholesterol (HDL-c), has been considered as a novel indicator revealing the status of lipid metabolism [8]. Robust correlations have as well been established between AIP and multiple CVD risk factors, which involved diabetes, hypertension, metabolic disorders etc., emphasizing the pivotal role of AIP in predicting pathologic condition of cardio-cerebrovascular system [9–11].Subsequently, its association with CVDs has been established [12, 13]. An elevation of AIP has been demonstrated to be predictive of higher incidence of coronary artery disease, cerebrovascular stenosis, myocardial infarction (MI), stroke, and major adverse cardiovascular events (MACEs) [14–19].

In spite of the notable predictive value of AIP for CVDs, there is considerable variation of AIP on account of various lifestyle and medication. A single measurement of blood lipid parameters might not reflect an individual’s long-term lipid metabolic status. Therefore, calculating cumulative AIP holds greater significance for predicting an individual’s cardiovascular risk. However, there are currently very few studies concerning this topic.

To address the gaps of previous research, we prospectively examined the association between cumulative AIP and the risk of cardiovascular outcomes in a community-based population.

## Methods

### Study design and population

Data for this study were taken from a community-based cohort centering on atherosclerosis, which was established in Gucheng and Pingguoyuan communities of Shijingshan District, Beijing, China. Initially, a total of 9540 residents (≥ 40 years old) were enrolled from December 2011 to April 2012. Target subjects were then invited to participate in onsite survey during May to July 2014. Detailed cohort information has been published elsewhere [20].

The endpoint data sources of this study were obtained by matching the identity information of the research subjects with external databases, including the national mortality surveillance system and Beijing inpatient medical record home page system. The cutoff date for obtaining subject information in the study was December 31, 2021. Disease codes were based on the 6-digit (extension code) format of the 10th Revision of the International Classification of Diseases (ICD-10), and death codes were based on the 4-digit (sub-level) format of the ICD-10 (details in supplementary Table 1).

In this analysis, the exposure variable was calculated as the average of that in 2012 and 2014, and regarding other variables, data obtained in 2014 were defined as baseline level. Ultimately, a total of 3820 subjects with complete triglyceride and HDL-c data in both 2012 and 2014 as well as endpoint data were included in the analysis.

Ethics committee approval was obtained from Peking University First Hospital for all study procedures. We obtained written informed consent from all participants.

### Data collection, variables and definitions

#### Questionnaire

All participants were interviewed by trained researchers using a standard questionnaire, which was used for the collection of demographic characteristics, lifestyle, medical histories (hypertension, diabetes mellitus, dyslipidemia, coronary heart disease, stroke etc.) and medications (antihypertensive drugs, hypoglycemic drugs and lipid-lowering drugs etc.).

#### Anthropometry

Anthropometric characteristics were measured by trained personnel according to a standard protocol. Weight and height were measured to the nearest 0.1 kg and 0.1 cm. Blood pressure (BP) was measured three times consecutively, with a 2-minute interval between each measurement using an Omron HEM-7117 electronic sphygmomanometer and the average value was used. Body mass index (BMI) was calculated by weight (kg) divided height square (m^2^).

#### Laboratory tests

Venous blood samples were obtained from participants after overnight fasting. The biological markers, which included total cholesterol (TC), TG, low-density lipoprotein cholesterol (LDL-c), HDL‐c, fasting blood glucose (FBG), were measured using a Hitachi 7180 Automatic Analyzer (Tokyo, Japan). Enzymatic measurement of plasma serum creatinine (Scr, µmol/L) was performed. The Chronic Kidney Disease Epidemiology Collaboration’s algorithm was used to get the estimated glomerular filtration rate (eGFR) [21].

#### Definition of medical history, smoking and alcohol drinking

Hypertension was defined as a self-reported history of hypertension or taking antihypertensive drugs or systolic BP ≥ 140 mm Hg, and/or diastolic BP ≥ 90 mm Hg. Diabetes mellitus was defined as a self‐reported history of diabetes or use of hypoglycemic drugs or FBG ≥ 7.0 mmol/L, and/or oral glucose tolerance test (OGTT) ≥ 11.1 mmol/L. Dyslipidemia was defined as a self‐reported history of dyslipidemia or any lipid parameter abnormality (LDL‐c ≥ 3.37 mmol/L or TG ≥ 1.70 mmol/L or TC ≥ 5.18 mmol/L or HDL‐c < 1.04 mmol/L), or the use of lipid‐lowering drugs. Cardiovascular disease was defined as a self‐reported history of stroke or coronary heart disease. The term “current smoking” was defined as smoking one cigarette a day for at least 6 months. The term “current drinking” was defined as consuming alcohol once every week for at least 6 months.

#### Definition of exposure and outcomes

AIP was calculated as the logarithmically transformed ratio of TG to HDL-c expressed in mmol/L. In the analysis, we used cumulative AIP, which is the average value of AIP in 2012 and 2014. The primary endpoint of the present study was MACE, which is a composite endpoint in combination of stroke, MI and cardiovascular mortality. The secondary endpoints comprised stroke, MI, and cardiovascular mortality as individual endpoints.

### Statistical analysis

Continuous variables were presented as the means and standard deviations (SDs), while categorical variables were expressed as numbers and percentages. Baseline characteristics were stratified by cumulative AIP values. Normally distributed variables were compared using one-way ANOVA while skewed distributed variables were compared using Kruskal Wallis test. Categorical variables were compared using the Pearson chi-square test or Fisher’s exact test. Time-to-event curves were estimated with the use of the Kaplan-Meier method. Cumulative AIP was analyzed as a continuous variable and then divided into quartiles, with the lowest quartile as the reference class. Multivariate Cox regression was used to assess the relationship between cumulative AIP and clinical outcomes. Trend tests were calculated by transforming cumulative AIP quartiles into a continuous variable. In the stratified analysis, possible modifications of the association between cumulative AIP and clinical outcomes were assessed for variables including age (< 65 vs. ≥65 years), sex (male vs. female), BMI (< 24 kg/m^2^ vs. ≥24 kg/m^2^), smoking (current smoking vs. non-current smoking), cardiovascular disease (no vs. yes), hypertension (no vs. yes), diabetes mellitus (no vs. yes), LDL-c (< 3.4 mmol/l vs. ≥3.4 mmol/l). Multivariate Cox regression models were used in the analysis of interaction.

The statistical analyses of this study were performed via R, version 4.2.0 (R Foundation) and EmpowerStats (http://www.empowerstats.com, X&Y Solutions, Inc., Boston, MA). The level of statistical significance was set at *p* < 0.05.

## Results

### Characteristics of the study participants

The baseline characteristics of the study subjects were listed in Table 1. A total of 3820 participants with a mean (SD) age of 59.1 (8.7) years, 36.1% of them were male, were ultimately included. Participants were divided into four groups based on cumulative AIP quartiles. Gender, BMI, current smoking and drinking status, cardiovascular disease, hypertension, diabetes mellitus, dyslipidemia, medications (anti-hypertensive drugs, lipid-lowering drugs), lipid levels (TC, LDL-c, HDL-c, TG) and eGFR were significantly different among four groups.

**Table 1:** Baseline characteristics of the study participants

Variables	Total (*N* = 3820)	Cumulative AIP quartiles				*p* value
		Q1(<-0.2)	Q 2(-0.2-<0.0)	Q3(0.0-<0.2)	Q4(≥ 0.2)	
Male, N (%)	1379 (36.1)	266 (27.9)	324 (33.9)	373 (39.1)	416 (43.6)	< 0.001
Age, year	59.1 (8.7)	58.7 (8.9)	59.4 (8.6)	59.8 (8.8)	58.7 (8.3)	0.011
BMI, kg/m^2^	26.0 (3.5)	24.2 (3.3)	25.7 (3.3)	26.8 (3.5)	27.2 (3.2)	< 0.001
Current smoking, N (%)	616 (16.1)	92 (9.6)	122 (12.8)	164 (17.2)	238 (25.1)	< 0.001
Current drinking, N (%)	535 (14.0)	118 (12.4)	125 (13.1)	137 (14.3)	155 (16.4)	0.066
Medical history, N (%)						
Hypertension	1774 (46.4)	337 (35.3)	418 (43.8)	489 (51.2)	530 (55.5)	< 0.001
Diabetes mellitus	780 (20.4)	144 (15.1)	177 (18.6)	206 (21.6)	253 (26.6)	< 0.001
Dyslipidemia	2908 (76.1)	451 (47.2)	679 (71.1)	832 (87.1)	946 (99.1)	< 0.001
Medications, N (%)						
Antihypertensive drugs	997 (26.1)	192 (20.1)	233 (24.4)	286 (29.9)	286 (30.2)	< 0.001
Antidiabetic drugs	364 (9.5)	69 (7.2)	95 (9.9)	91 (9.5)	109 (11.5)	0.016
Lipid-lowering drugs	369 (9.7)	66 (6.9)	86 (9.0)	110 (11.5)	107 (11.3)	0.001
Laboratory tests						
TC, mmol/L	5.0 (1.0)	4.9 (0.9)	5.0 (1.0)	5.0 (1.0)	5.1 (1.0)	< 0.001
LDL-c, mmol/L	2.9 (0.8)	2.8 (0.7)	3.1 (0.8)	3.1 (0.8)	2.8 (0.8)	< 0.001
HDL-c, mmol/L	1.2 (0.3)	1.5 (0.3)	1.3 (0.2)	1.1 (0.2)	1.0 (0.2)	< 0.001
TG, mmol/L#	1.4 (1.0, 2.0)	0.8 (0.7, 1.0)	1.3 (1.0, 1.5)	1.6 (1.4, 2.0)	2.6 (2.1, 3.4)	< 0.001
eGFR, mL/(min·1.73 m2 )	73.8 (11.8)	75.3 (11.3)	74.2 (11.8)	73.0 (12.0)	72.8 (11.7)	< 0.001

Data is presented with number of participants (percentage) or mean (SD)

#Presented as median (25th, 75th percentile)

*p* < 0.05 indicate significant difference between or across groups

Abbreviations: AIP, atherogenic index of plasma; BMI, body mass index; TG, triglyceride; TC, total cholesterol; LDL-c, low-density lipoprotein cholesterol; HDL-c, high-density lipoprotein cholesterol; eGFR, estimated glomerular filtration rate

### Association between cumulative AIP and clinical outcomes

#### Association between cumulative AIP and MACE

**Fig. 1:**
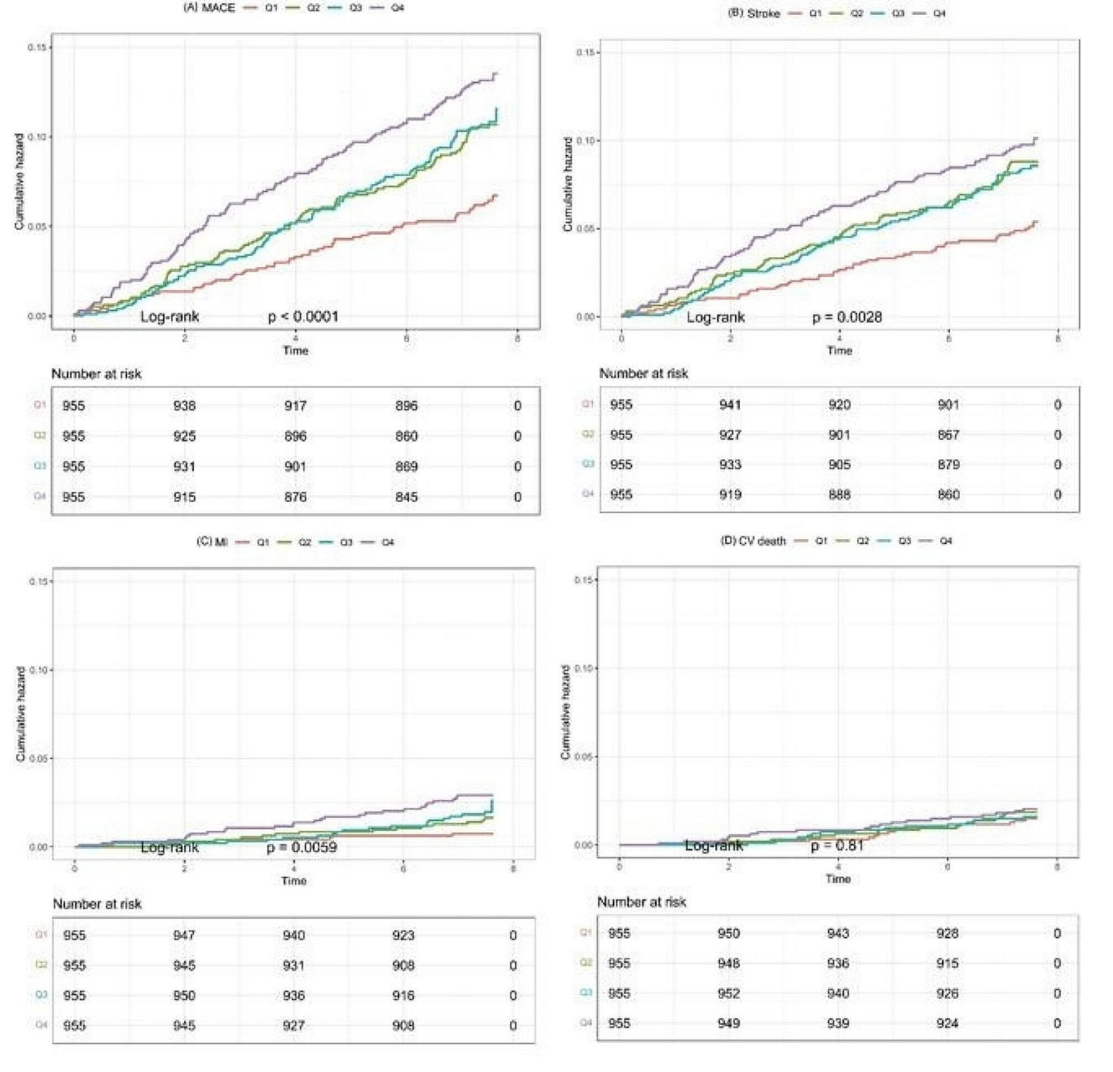
Kaplan-Meier estimates for outcome events. Panels present the Kaplan-Meier curves for (A) MACE, (B) stroke, (C) myocardial infarction and (D) CV death in 3 820 participants stratified into four groups according to cumulative AIP levels. Abbreviations AIP, atherogenic index of plasma; MACE, major adverse cardiac event; CV, cardiovascular

371 (9.7%) subjects developed MACE during a median follow-up period for 7.5 years. Time-to-event analyses were shown in Fig. 1(A). Kaplan-Meier curve suggested that higher cumulative AIP was associated with increased risk of MACE (logrank, *p* < 0.001).

Table 2 showed the results from the Cox regression analyses between cumulative AIP and different clinical outcomes. Continuous cumulative AIP was positively correlated with MACE in crude model (model 1), model 2 which adjusted for demographic covariates and model 3 which further adjusted for more confounders including age, gender, BMI, current smoking status, cardiovascular disease, hypertension, diabetes mellitus, antihypertensive drugs, antidiabetic drugs, lipid-lowering drugs, LDL-c, and eGFR (*p* < 0.05 for each model). When cumulative AIP was divided into quartiles, compared with Q1, the risk of MACE for subjects in Q2, Q3 and Q4 were significantly higher in unadjusted model 1 (*p* < 0.001). Nevertheless, only participants in Q2 and Q4 were significantly associated with increased risk of MACE in model 2 (Q2: HR = 1.53, 95%CI: 1.10–2.12, *p* = 0.011; Q4: HR = 2.01, 95%CI: 1.45–2.77, *p* < 0.001) and model 3 (Q2: HR = 1.45, 95%CI: 1.04–2.01, *p* = 0.028; Q4: HR = 1.76, 95%CI: 1.27–2.44, *p* < 0.001). It remained consistent that the risk manifested a significant upward trend across groups as cumulative AIP quartiles increased (*p* for trend < 0.05 in three models).

**Table 2:** Association of cumulative AIP and clinical outcomes

Cumulative AIP	Events number, *N* (%)	Model 1*	*p* value	Model 2#	*p* value	Model 3†	*p* value
		HR (95%CI)		HR (95%CI)		HR (95%CI)	
**MACE**							
Continuous	371 (9.7)	2.05 (1.46, 2.88)	< 0.001	2.05 (1.42, 2.96)	< 0.001	1.75 (1.19, 2.57)	0.004
Quartiles							
Q1	60 (6.3)	*Ref.*		*Ref.*		*Ref.*	
Q2	95 (9.9)	1.63 (1.18, 2.25)	0.003	1.53 (1.10, 2.12)	0.011	1.45 (1.04, 2.01)	0.028
Q3	98 (10.3)	1.66 (1.21, 2.30)	0.002	1.47 (1.05, 2.04)	0.023	1.32 (0.95, 1.85)	0.100
Q4	118 (12.4)	2.05 (1.50, 2.80)	< 0.001	2.01 (1.45, 2.77)	< 0.001	1.76 (1.27, 2.44)	< 0.001
*p* for trend			< 0.001		< 0.001		0.002
**Stroke**							
Continuous	293 (7.7)	1.76 (1.20, 2.59)	0.004	1.75 (1.15, 2.66)	0.009	1.51 (0.98, 2.33)	0.063
Quartiles							
Q1	48 (5.0)	*Ref.*		*Ref.*		*Ref.*	
Q2	79 (8.3)	1.69 (1.18, 2.42)	0.004	1.58 (1.10, 2.28)	0.013	1.51 (1.05, 2.18)	0.027
Q3	77 (8.1)	1.63 (1.14, 2.34)	0.008	1.44 (1.00, 2.09)	0.052	1.33 (0.91, 1.94)	0.135
Q4	89 (9.3)	1.91 (1.35, 2.72)	< 0.001	1.87 (1.30, 2.70)	< 0.001	1.67 (1.15, 2.41)	0.006
*p* for trend			0.001		0.003		0.022
**MI**							
Continuous	68 (1.8)	4.14 (1.93, 8.85)	< 0.001	3.70 (1.62, 8.44)	0.002	3.61 (1.46, 8.95)	0.006
Quartiles							
Q1	7 (0.7)	*Ref.*		*Ref.*		*Ref.*	
Q2	15 (1.6)	2.17 (0.88, 5.32)	0.091	1.93 (0.78, 4.77)	0.154	1.74 (0.70, 4.32)	0.232
Q3	19 (2.0)	2.72 (1.14, 6.48)	0.024	2.16 (0.89, 5.24)	0.088	1.81 (0.74, 4.42)	0.194
Q4	27 (2.8)	3.91 (1.70, 8.98)	0.001	3.32 (1.41, 7.85)	0.006	2.80 (1.18, 6.67)	0.020
*p* for trend			0.001		0.004		0.017
**Cardiovascular mortality**							
Continuous	65 (1.7)	1.43 (0.62, 3.28)	0.404	2.62 (1.07, 6.40)	0.035	1.84 (0.70, 4.83)	0.217
Quartiles							
Q1	14 (1.5)	*Ref.*		*Ref.*		*Ref.*	
Q2	17 (1.8)	1.23 (0.61, 2.49)	0.569	1.44 (0.70, 2.96)	0.322	1.10 (0.52, 2.33)	0.793
Q3	15 (1.6)	1.07 (0.52, 2.22)	0.850	1.25 (0.59, 2.66)	0.563	0.96 (0.44, 2.09)	0.921
Q4	19 (2.0)	1.36 (0.68, 2.72)	0.379	2.17 (1.04, 4.52)	0.039	1.50 (0.71, 3.15)	0.286
*p* for trend			0.470		0.068		0.342

Data is presented with number of participants (percentage) or mean (SD)

*p* < 0.05 indicate significant difference between or across groups

*Model 1 was a crude model

#Model 2 adjusted for age, gender and BMI

†Model 3 adjusted for age, gender, BMI, current smoking status, cardiovascular disease, hypertension, diabetes mellitus, antihypertensive drugs, antidiabetic drugs, lipid-lowering drugs, LDL-c, and eGFR

*Abbreviations*: AIP, atherogenic index of plasma; BMI, body mass index; TG, triglyceride; TC, total cholesterol; LDL-c, low-density lipoprotein cholesterol; HDL-c, high-density lipoprotein cholesterol; eGFR, estimated glomerular filtration rate; MACE, major adverse cardiovascular events; MI, myocardial infarction

#### Association between cumulative AIP and stroke

Among subjects with MACE, a total of 293 individuals experienced stroke. The Kaplan-Meier curve demonstrated that higher cumulative AIP was also associated with increased risk of stroke (logrank, *p* = 0.003, Fig. 1 [B])

The results from Cox regression analyses, as manifested in Table 2, indicated a significantly positive association between cumulative AIP and stroke in model 1 (HR = 1.76, 95%CI: 1.20–2.59, *p* = 0.004) as well as model 2 (HR = 1.75, 95%CI: 1.15–2.66, *p* = 0.009), and the correlation remained marginal significant in model 3 (HR = 1.53, 95% CI: 1.00-2.35, *p* = 0.063). Similar to results obtained regarding MACE, after divided into quartile, compared with Q1, the risk of stroke for subjects in Q2, and Q4 were significantly higher after adjusting for all covariates in model 3 (Q2: HR = 1.52, 95% CI: 1.05–2.19, *p* = 0.025; Q4: HR = 1.69, 95%CI: 1.17–2.45, *p* = 0.005). The risk also significantly increased across groups (*p* for trend = 0.022)

#### Association between cumulative AIP and MI

68 participants developed myocardial infarction during follow-up and Kaplan-Meier curve revealed that higher cumulative AIP was as well associated with increased risk of MI (logrank, *p* = 0.006, Fig. 1[C]).

As was shown in Table 2, continuous cumulative AIP was significantly correlated with myocardial infarction in all models (*p* < 0.01). Every 1 increment of cumulative AIP brought out 256% increased risk of MI even after adjusting all confounders in model 3 (HR = 3.61, 95%CI: 1.46–8.95, *p* = 0.006). When divided into quartiles, subjects in the group with the highest cumulative AIP have 1.82-fold higher risk of suffering MI compared to those in the group with the lowest cumulative AIP in model 3 (HR = 2.82, 95%CI: 1.18–6.72, *p* = 0.019). The trend test was significant as well (*p* for trend = 0.017)

#### Association between cumulative AIP and cardiovascular mortality

A total of 65 subjects suffered from cardiovascular death. Kaplan-Meier curve showed that cumulative AIP and cardiovascular mortality was not correlated (logrank, *p* = 0.810, Fig. 1[D]).

In Cox analyses, regardless of being treated as a continuous variable or divided into quartiles, cumulative AIP was not correlated with the occurrence of cardiovascular mortality, as shown in Table 2 (all *p* values and *p* for trend > 0.05)

### Assessment of interaction

**Fig. 2:**
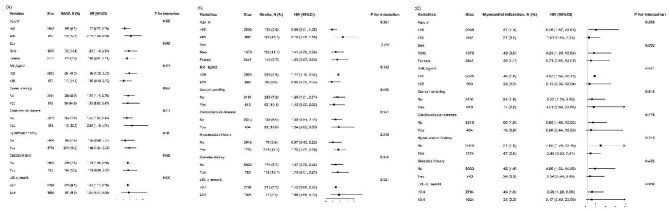
Subgroup analysis on the association between cumulative AIP and cardiovascular outcomes Hazard ratios (HRs) have been fully adjusted for age, gender, BMI, current smoking status, cardiovascular disease, hypertension, diabetes mellitus, antihypertensive drugs, antidiabetic drugs, lipid-lowering drugs, LDL-c, and eGFR. *p* < 0.05 indicate significant difference Abbreviations: AIP, atherogenic index of plasma; MACE, major adverse cardiac event; BMI, body mass index; LDL-c, low-density lipoprotein cholesterol; eGFR, estimated glomerular filtration rate

Figure 2 presented the results of modification effects between cumulative AIP and different clinical endpoints in various subgroups. There was no interaction between cumulative AIP and MACE or myocardial infarction. An interaction was only found between cumulative AIP and stroke. The correlation of cumulative AIP and the incidence of stroke was more pronounced in the elderly (HR: 0.89 vs. 2.41 for the age groups < 65 years and ≥ 65 years, *p* for interaction = 0.018). There was no significant modification effect in other subgroups, including gender, BMI, current smoking status, cardiovascular disease, hypertension, diabetes mellitus and LDL-c (all *p* for interaction > 0.05)

## Discussion

In this large community-based cohort study, we observed a notable association between higher cumulative AIP and an elevated risk of MACE, as well as its sub-endpoints, stroke and myocardial infarction independent of traditional cardiovascular risk factors. Further, subgroup analysis revealed that the predictive capacity of cumulative AIP for stroke was particularly pronounced in the elderly population. These findings underscore the potential utility of cumulative AIP as a tool for identifying cardiovascular event risk within the general population.

A substantial amount of studies has confirmed the predictive value of AIP for the risk of cardiovascular events [22–27]. However, AIP in these studies was calculated based on single measurement of blood lipid profile. Researchers have found that single measurement of blood lipid parameters may have been influenced by various factors such as diet and medication, thus leading to high probable variability of these parameters, and may not fully reflect a patient’s long-term lipid exposure. Therefore, to address the limitations of baseline AIP in lack of representativeness of a long-term AIP level, cumulative AIP has been proposed, drawing increasing attention.

In previous studies, cumulative AIP was reported to be associated with an increased the risk of CVD risk factors, such as type 2 diabetes mellitus [28, 29], as well as a higher incidence of CVD and cardiovascular events [17, 30, 31]. In a cohort study of 54 123 participants (Kailuan Study), Zheng et al. 2023 [30] reported that higher cumulative AIP was associated with a higher risk of ischemic stroke. The calculation of cumulative AIP was by using a weighted sum of the mean AIP values for each time interval and then normalized to the total duration of exposure (2006–2010), which closely resembles the calculation approach adopted in our study in terms of both concept and timespan. Similarly, Zhang et al. 2023 [31] revealed that long-term cumulative exposure to AIP increased the risk of myocardial infarction in the same cohort enrolling 54,440 participants.

Existing explorations have focused solely on single endpoints (e.g., ischemic stroke and myocardial infarction, respectively), whereas our research may have stood out since we have considered the composite endpoint of MACE, which could better reflect the comprehensive risk of cardiovascular events. Additionally, there are some differences in the demographic characteristics of the populations studied in our research compared to previous studies concerning cardiovascular events. Participants recruited in Kailuan cohort are primarily coal miners, whereas our cohort comprises residents from communities in urban areas of Beijing. Significant disparities exist between the two cohorts in terms of living environments, exposure to air pollutants, educational levels, economic status and medical care. Results from our cohort might demonstrate better representativeness for the general population in northern China.

Potential explanations have been proposed regarding the mechanism underlying the predictive value of AIP. AIP serves as a composite biomarker reflecting the balance between TG and HDL-c Impaired TG metabolism has been recognized as one of the residual risk factors beyond LDL-c level and thus has been closely linked to the development of CVD and subsequent poor clinical outcomes [32–34]. Meanwhile, HDL-c long plays a crucial role in cholesterol clearance [35]. Previous explorations have demonstrated that an elevated TG/HDL-c ratio correlates with high concentrations of proatherogenic lipoprotein subclasses and the presence of small dense LDL particles [36, 37]. Furthermore, earlier studies have established a close association between AIP and lipoprotein particle size, particularly an inverse correlation with LDL particle size [38]. LDL particle size has been associated with the progression of CVD and incidence of cardiovascular events, with a predominance of small, dense LDL being an emerging cardiovascular risk factor [39]. Therefore, higher AIP indicates smaller LDL particle size, and therefore serving as a surrogate marker of atherogenicity [38].

Another finding of this study was that the correlation of cumulative AIP and stroke was more pronounced among elderly individuals. The explanation might be that, in the elder population, higher cumulative AIP indicates a prolonged history of chronic long-term exposure to an atherosclerotic condition, rendering these individuals more susceptible to experiencing a stroke event. Moreover, the elder population is more likely to have been complicated with other cardiovascular risk factors, which may involve hypertension, diabetes, hyperlipidemia etc., consequently, this particular population may find itself naturally in a more vulnerable state. Given these circumstances, the elder population would be more susceptible to cardiovascular events rather than younger individuals under the same exposure to AIP.

This study bears several limitations. Firstly, diet and physical activities, both potential confounding factors that may influence the course of atherosclerosis, were not recorded in this cohort, which might have induced potential bias during risk assessment. Secondly, the values of adjusted covariates were solely collected at baseline, and their values may have changed over time. Additionally, residual or unknown confounding factors cannot be entirely excluded, thus leading to a potential influence on the association of cumulative AIP with cardiovascular outcomes. Thirdly, while our cohort represents the general population in northern China, the generalizability of our findings to other countries or ethnic groups warrants further validation. Fourthly, the detailed mechanism explaining the atherogenic effect of AIP has not been fully explored, necessitating further experimental and clinical studies for more comprehensive elucidation.

## Conclusions

In this large community-based cohort study, we demonstrated that a higher cumulative AIP was significantly associated with an increased risk of MACE, stroke and myocardial infarction independent of traditional cardiovascular risk factors, and the association has been particularly pronounced in the elderly with respect to stroke. These findings underscore the potential of cumulative AIP as a predictive marker for multiple cardiovascular events within the general population. More large-scale population-based investigations are needed to further validate our findings.

### Limitations

One notable limitation of our study is the lack of detailed data on lifestyle factors such as diet and physical activity, which might influence AIP levels. While our research primarily focuses on the cumulative AIP values over a two-year period and their impact on cardiovascular events, the absence of specific information on dietary and physical activity trends during this period limits our ability to fully account for their potential effects on AIP and cardiovascular outcomes. Future studies should aim to incorporate comprehensive lifestyle data to provide a more holistic understanding of the relationship between AIP levels and cardiovascular health. We acknowledge this gap and recommend that subsequent research address these factors to enhance the insights provided by such studies.

### Electronic supplementary material

Below is the link to the electronic supplementary material.

Supplementary Material 1

## Data Availability

No datasets were generated or analysed during the current study.

## Abbreviations

AIP: Atherogenic index of plasma
CVD: Cardiovascular disease
MACE: Major adverse cardiac event
MI: Myocardial infarction
TG: Triglyceride
HDL-c: High-density lipoprotein cholesterol
LDL-c: Low-density lipoprotein cholesterol
TC: Total cholesterol
BP: Blood pressure
BMI: Body mass index
FBG: Fasting blood glucose
OGTT: Oral glucose tolerance test
eGFR: Estimated glomerular filtration rate
