# Supplementary material for: The predictive value of cumulative atherogenic index of plasma (AIP) for cardiovascular outcomes: a prospective community-based cohort study
Source: Cardiovasc Diabetol. 2024 Jul 18;23:264. doi: 10.1186/s12933-024-02350-8 (PMC11264486; doi:10.1186/s12933-024-02350-8)
Supplement: Supplementary file 1 — Supplementary Material 1 [file 12933_2024_2350_MOESM1_ESM.docx]

Supplementary Table 1. Sources and definitions of endpoint events with ICD-10 codes.

| Endpoint events | Sources of data | Codes |
| --- | --- | --- |
| Cardiovascular death | National mortality surveillance system | I00-I99 |
| Myocardial infarction | Beijing inpatient medical record home page system | Acute ST-segment elevated myocardial infarction (I21.001-006, I21.101-105, I21.201-211, I21.213-230, I21.301, I21.304, I22.001-003, I22.101-103, I22.801-818), Acute non ST-segment elevated myocardial infarction (I21.401-404), other myocardial infarction (I21.302, I21.303, I21.305-308, I21.901, I21.902, I21.907, I21.910, I21.911, I22.901) |
|  | National mortality surveillance system | I21.0, I21.1, I21.2, I21.3, I21.4, I21.9, I22.0, I22.1, I22.8, I22.9 |
| Stroke | Beijing inpatient medical record home page system | Ischemic stroke (I63), hemorrhagic stroke (I60-I61), other stroke (I64) |
|  | National mortality surveillance system | I60, I61, I63, I64 |
